# Non-classical 11β-hydroxylase deficiency caused by compound heterozygous mutations: a case study and literature review

**DOI:** 10.1186/s13048-018-0450-8

**Published:** 2018-09-17

**Authors:** Dongdong Wang, Jiahui Wang, Tong Tong, Qing Yang

**Affiliations:** Obstetrics and Gynecology Department of Shengjing hospital, China Medical University, Shenyang, 110001 People’s Republic of China

**Keywords:** 11β-hydroxylase deficiency, Genetic testing, Hypertension, Protein function prediction

## Abstract

**Background:**

11β-hydroxylase deficiency (11OHD) is extremely rare, and reports of non-classical 11OHD are even rarer. Non-classical 11OHD usually presents as premature adrenarche, hyperandrogenism, menstrual disorders, and hypertension. Because the symptoms of non-classical 11OHD are mild, delayed diagnosis or misdiagnosis as polycystic ovary syndrome or primary hypertension is common.

**Case presentation:**

This paper introduces a case of a young female patient presenting hypertension and menstrual disorders. Laboratory examination revealed increased androgen levels, mild adrenal hyperplasia, mild left ventricular hypertrophy, and mild sclerosis of the lower limb arteries. 11OHD was confirmed by genetic testing, and the patient was found to carry compound heterozygous mutations in *CYP11B1* (c.583 T > C and c.1358G > A). The mutation Y195H is located in exon 3 and has not been reported previously. In silico studies indicated that this mutation may cause reduced enzymatic activity. After treatment with hydrocortisone and spironolactone, blood pressure was brought under good control, and menstruation returned to normal. We also conducted a retrospective review of previously reported cases in the literature (over 170 cases since 1991).

**Conclusions:**

Early diagnosis of non-classical 11OHD is difficult because its symptoms are mild. The possibility of this disease should be considered in patients with early-onset hypertension, menstrual disorders, and hyperandrogenism to provide early treatment and prevent organ damage due to hypertension and hyperandrogenism. *CYP11B1* mutations are known to be race-specific and are concentrated in exons 3 and 8, of which mutations in the former are mostly associated with non-classical 11OHD, whereas mutations in the latter are mostly found in classical 11OHD, characterized by severe loss of enzymatic activity.

## Background

Congenital adrenal hyperplasia (CAH) is a common genetic endocrine metabolic disorder, of which 21-hydroxylase deficiency (21OHD) is the most common type, accounting for 90–99% of all CAH cases. The second most common type of CAH is 11β-hydroxylase deficiency (11OHD), which accounts for 0.2–8% of cases [1]. Steroid 11b-hydroxylase defects lead to reduced conversion of 11-deoxycortisol (S) and 11-deoxycorticosterone (DOC) to cortisol and corticosterone, thereby leading to accumulation of the two steroid precursors mentioned above. In addition, an increase in metabolic products towards sex steroids corresponds to the typical clinical presentation including low renin hypertension, hypokalemia, hyperandrogenemia, and genital ambiguity in affected females. Current reports in the literature relevant to 11βOHD mostly involve classical 11OHD, and there are relatively few reports on non-classical 11OHD because of its mild symptoms.

## Case presentation

The patient was a 23-year-old female (46, XY karyotype) diagnosed with hypertension (180/120 mmHg) since age 14 and a BMI of 20.8 kg/m^2^. There was no obvious masculinization, and her parents stated that there were no obvious abnormalities in vulva development at birth. Antihypertensive drug therapy (nifedipine sustained-release tablets) had been taken continuously, and blood pressure was controlled to 130–140/80–90 mmHg. The patient sought treatment at our hospital due to menstrual disorders. The patient is the only child of non-consanguineous healthy parents from Northeast China. The study was approved by the ethics committees of China Medical University, and informed consent was obtained from the patient and her parents.

### Clinical examination and testing

Imaging examinations included an ultrasonic cardiogram, a colour Doppler ultrasound of the carotid artery and lower limb arteries, a pelvic colour Doppler ultrasound (SSA660A, Toshiba), and a contrast-enhanced adrenal computer tomography scan (16-slice computer tomography machine, GE Lightspeed). Laboratory tests included measurements of serum potassium, natrium, testosterone, free testosterone, androstenedione, dehydroepiandrosterone sulphate, adrenocorticotropic hormone, cortisol, 17-hydroxyprogesterone, renin, and aldosterone using chemiluminescence immunoassays and biochemical assays.

### Genetic analysis

Peripheral blood samples from the patient and her parents were collected for gene analysis. Direct sequencing was performed on all the exons and the exon–intron boundaries of CYP21A2 (NM_000500) and CYP11B1 (NM_000497.3).

### In silico analysis

PolyPhen-2 (http://genetics.bwh.harvard.edu/pph2, Protein ID for CYP11B1 is NP_000488.3 or P15538) and SIFT/Provean (http://sift.jcvi.org/) were used to predict whether an amino acid substitution affects protein function. The alignment in CYP11 families was performed using CYP11B1 sequences from different species and other human steroidogenic P450 cytochromes. PolyPhen-2 and DNAMAN software was used for multiple amino acid sequence alignment. CYP11B2 (PDB entry: 4DVQ.A), which shares 93.6% sequence identity with CYP11B1, was selected as the template for model building of CYP11B1. The structural representations were generated using PyMOL 2.0.6.

## Results

### Clinical characteristics and serum hormone levels

The patient’s blood pressure at admission was 140/100 mmHg. The laboratory examination results are shown in Table 1. Blood potassium was normal, and androgen levels were increased. Adrenal CT indicated mild hyperplasia of both adrenal glands. Echocardiography indicated mild hypertrophy and slight enlargement of the left ventricle. Vascular ultrasound indicated mild sclerosis of the arteries in the lower limbs. Ultrasound of the uterine adnexa did not reveal any abnormality.

**Table 1:** Summary of laboratory data for the affected subject with steroid 11β-hydroxylase deficiency

Parameter	Result		Reference range
	Basal level	3 months after treatment with hydrocortisone	
ACTH (pg/ml)	48.9	16.59	7.2–63.3
Cortisol (nomol/L)	322	391	171–536
Aldosterone (ng/ml)	0.13	0.12	0.07–0.30
Renin (ng/ml)	0.08	0.44	0.93–6.56
Serum K^+^ (mmol/L)	3.64	4.13	3.50–5.30
Serum Na^+^ (mmol/L)	141.2	139.2	137.0–147.0
Testosterone (nmol/L)	6.07	0.76	0.69–2.77
Androstendione (nmol/L)	> 35	6.8	2.09–10.82
DHEA-S (umol/L)	5.02	3.31	0.95–11.67
Free Testosterone (pmol/L)	42.27	13.24	0.77–33.03
Estradiol (pmol/L)	202	–	73.4–587
Progestogen (nmol/L)	4.67	–	0.64–3.6
17OHP (nmol/L)	11.6	–	0–30

*ACTH* Adrenocorticotropic Hormone, *K+* Potassium, *DHEA-S* Dehydroepiandrosterone sulfate, *17OHP* 17-hydroxyprogesterone

### Genetic analysis

**Fig. 1:**
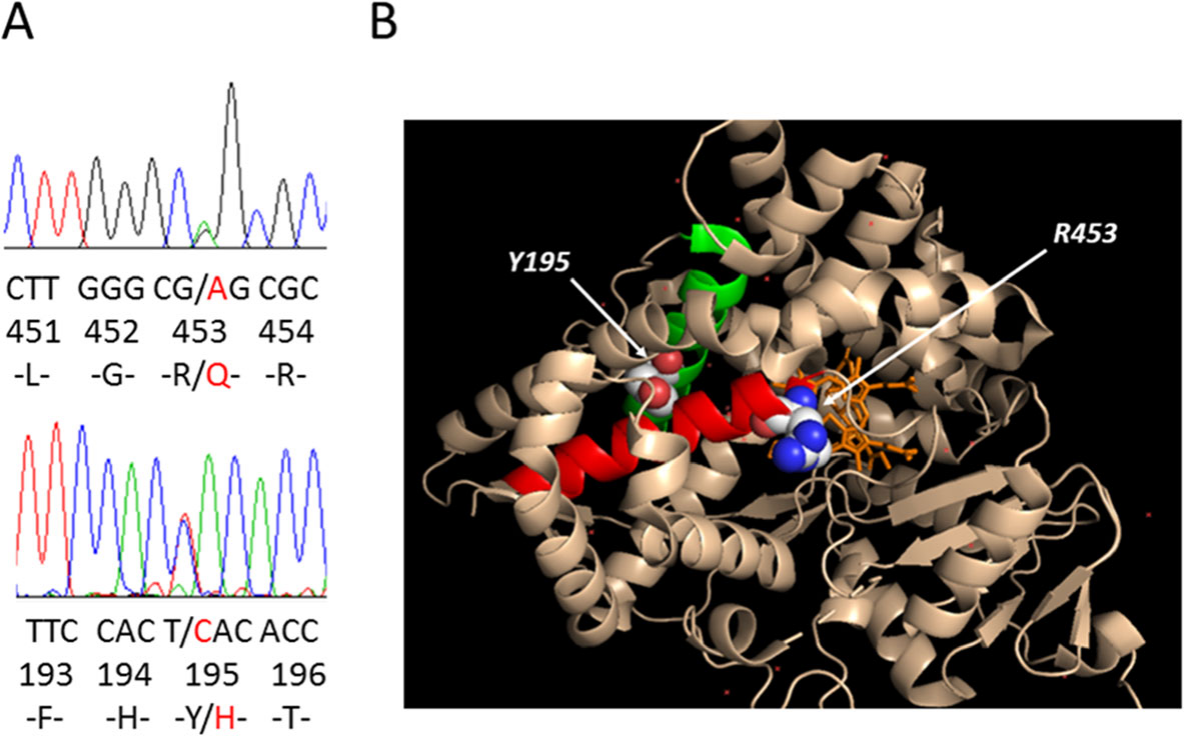
*CYP11B1* sequencing results and 3D molecular schematic representation of the mutation site. **a** Compound heterozygous mutation (c.583 T > C and c.1358G > A) that leads to the conversion of tyrosine at amino acid position 195 to histidine (Y195H) and arginine at amino acid position 453 to glutamine (R453Q). **b** Three-dimensional model structure of CYP11B1. Green, E helix; red, L helix. The side chains of amino acid residues Y195 (on the E helix) and R453 (on the L helix) are depicted

No mutations in CYP21A2 were found in the patient. Exons 3 and 8 of CYP11B1 harboured a compound heterozygous mutation (c.583 T > C and c.1358G > A) leading to the conversion of tyrosine at amino acid position 195 to histidine (Y195H) and arginine at amino acid position 453 to glutamine (R453Q) (Fig. 1a). Each parent of the patient carried one of these heterozygous mutations.

### Bioinformatics and in silico analysis of Y195H

Homology alignments indicate that the Tyr195 residue in CYP11B1 is highly conserved among different species, but compared to other human CYP family members, it is only the same in CYP11B2 (Table 2). In silico analysis by both PolyPhen-2 and SIFT/Provean predicted a pathogenic effect of the novel mutation Y195H. The amino acid residue Y195 is localized in the E helix (Fig. 1b).

**Table 2:** Homology alignments between CYP family members

Protein ID (UniProtKB/Swiss-Prot)	Sequence framing Tyr195
P15538 (HUMAN CYP11B1)	TLDVQPSIFH **Y** TIEASNLAL
F7GMV0 (*Macaca mulatta* CYP11B1)	TLDVQPSIFH **Y** TIEASNLAL
F6XJ24 (*Equus caballus* (Horse) CYP11B1)	TLDARPSIFH **Y** TIEASNLAL
P51663 (*Ovis aries* (Sheep) CYP11B1)	TLDIAPSVFR **Y** TIEASTLVL
Q29552 (*Sus scrofa* (Pig) CYP11B1)	TLDIKPSIFR **Y** TIEASNLVL
P15150 (*Bos taurus* (Bovine) CYP11B1)	TLDIAPSVFR **Y** TIEASNLVL
Q3TG86 (*Mus musculus* (Mouse) CYP11B1)	SMDFQSSVFN **Y** TIEASHFVL
P15393 (*Rattus norvegicus* (Rat) CYP11B1)	SINIQSNMFN **Y** TMEASHFVI
P19099 (HUMAN CYP11B2)	TLDVQPSIFH **Y** TIEASNLAL
P05108 (HUMAN CYP11A1)	SGDISDDLFR **F** AFESITNVI
P05093 (HUMAN CYP17A1)	IDNLSKDSLV **D** LVPWLKIFP
P08686 (HUMAN CYP21A2)	SLLTCSIICY **L** TFGDKIKDD
P11511 (HUMAN CYP19A1)	AESLKTHLDR **L** EEVTNESGY

### Intervention and outcome

After diagnosis of non-classical 11OHD was confirmed, the patient was given hydrocortisone twice a day (hydrocortisone 10 mg in the morning and 5 mg in the afternoon) and spironolactone (40 mg each day). Her blood pressure was brought under good control gradually (120/70 mmHg), and menstruation returned to normal with decreased androgen levels (Table 1).

## Discussion and conclusions

**Fig. 2:**
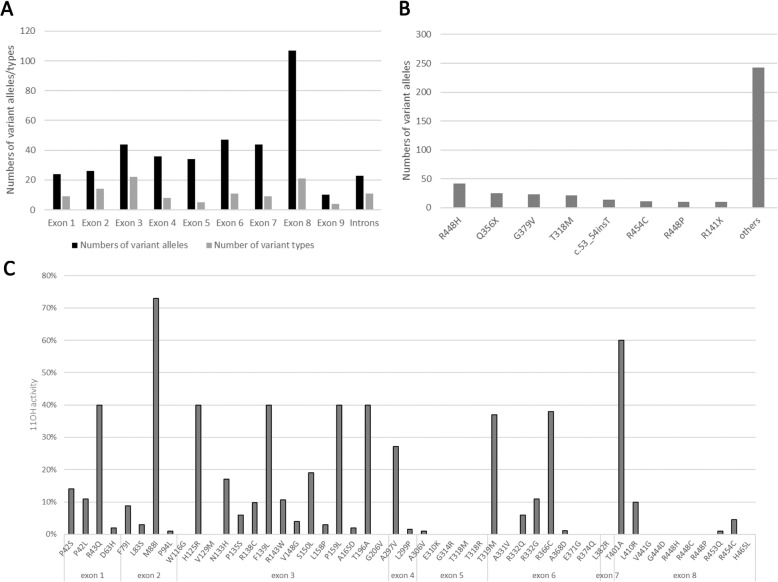
Characteristics of previously reported *CYP11B1* mutations in the literature. **a** Distribution of *CYP11B1* mutations. **b** The 8 most common types of mutations. **c** Effect of known mutation sites on 11OH enzymatic activity

11βOHD is an autosomal recessive genetic disease. Over 100 mutations of *CYP11B1* have been reported in the literature to date. There are more homozygous than compound heterozygous mutations (69.1% vs. 29.8%), and there are few single heterozygous mutations (1.1%), possibly because mutations in other exons have not yet been discovered [2, 3]. In the past, it was believed that mutations that cause 11βOHD are mostly concentrated in exons 2, 6, 7, and 8 [2, 4, 5]. However, our statistics revealed that most causative mutations are located in exons 3 and 8 (40%), and that the distribution in the other exons is actually more average. Patients carrying mutations in exon 8 account for the highest proportion among all patients, and patients carrying the R448H mutation are the most numerous (Fig. 2a) [5–12]. Because the highly conserved amino acid sequence near C450 is located in exon 8, the normal structure of this region is essential for maintaining normal enzymatic activity [2]. Thus, most point mutations in exon 8 result in severe reduction of enzymatic activity, thereby resulting in classical 11βOHD. Although there are many mutations in exon 3, most cause a partial reduction in enzymatic activity and thus result in non-classical 11βOHD (Fig. 2c) [5, 7, 13–23]. Thus, the probability of a mutation appearing in each exon is similar, but because the mutations reduce enzymatic activity to different degrees, different severities of clinical presentation are observed, and there is a high probability of misidentifying the disease for mutations in certain exons. The R448H mutation and 8 other mutations are the most frequently reported and account for approximately 40% of all cases (Fig. 2b). *CYP11B1* mutation shows significant race specificity. For example, the R448H mutation mentioned above is common among Moroccan Jews [6]. Tunisians often carry the two mutations, G379 V and Q356X [24], of which Q356X is also common among sub-Saharan Africans and African-Americans [2, 3, 24–26]. The T318 M mutation is most common among Yemenis [2, 27, 28], and some new mutations such as c.53_54insT, G206 V, W260X, R448P, and H465L are often found in Saudi Arabs [29–31]. The R454C mutation has only been reported among the Chinese [8, 10, 32].

The case reported in this paper is a patient carrying the compound heterozygous mutations Y195H and R453Q located in exons 3 and 8, respectively, which are mutation hotspots. Of these mutations, R453Q has been reported in one individual of European descent and 3 Chinese individuals [8, 23, 33], and is likely to be more common among the Chinese [33]. The residue R453 is located in the L-helix and is adjacent to the Cys-pocket motif. This domain is highly conserved in the P450 family of enzymes, and causes 11-hydroxylase activity to decrease to approximately 1% of the wild-type activity [23]. The Y195H mutation has not been reported previously. Y195 is located in the E helix. Homology alignments indicate that this amino acid sequence is relatively conserved, and predictions using PolyPhen-2 and SIFT/Provean indicate that this mutation may impair protein function. A mutation in the neighbouring amino acid T196 has been reported previously (T196A). This mutation can lead to a 30–50% loss in enzymatic activity and can result in non-classical 11OHD [15]. Three-dimensional structural models show that Y195 and T196 are located in the middle segment of the E helix and are close to L463-L464 in the L helix, which is involved in heme binding [34]. This suggests that Y195H and T196A are similar and may affect enzymatic activity by indirectly affecting the structure of the L helix, but to a small degree; it is thus a mutation that causes non-classical 11OHD.

Because the clinical presentation of non-classical 11OHD is atypical and highly variable, its prevalence may be underestimated and it may be misdiagnosed. Some patients may be misdiagnosed as having polycystic ovary syndrome because of the mildly elevated androgen levels [13, 16], and there are patients that only present with hypertension with mineralocorticoid features [15]. After the ACTH stimulation test, the degree of increase in 17-hydroxyprogesterone can be used to differentiate from non-classical 21OHD, but there is still no consensus on the diagnostic standards for non-classical 11OHD [4, 17, 21]. Baseline and stimulated 11-deoxycortisol measurements, 11β-hydroxylase activity assays, and urinary steroid profiling using LC-MS/MS are recommended to avoid missing the diagnosis of non-classical 11OHD [16, 35]. However, 11-deoxycortisol measurement and ACTH medications are currently unavailable in most Chinese hospitals, which hinders the diagnosis of 11OHD, especially non-classical 11OHD. As the cost of genetic diagnosis decreases, genetic testing of *CYP11B1* in suspected patients may make the diagnosis of non-classical 11OHD convenient and accurate.

Non-classical 11OHD patients may develop hypertension [14, 16], but this is highly variable, and its incidence and extent are still not clear [35]. The patient in our case developed severe hypertension at an early age, but because her diagnosis was never confirmed, antihypertensive therapy was irregular and she developed left ventricular myocardial hypertrophy and arteriosclerosis-like changes in the lower limbs, indicating the importance of early diagnosis and antihypertensive therapy. The use of mineralocorticoid receptor antagonists such as spironolactone or eplerenone for antihypertensive therapy is recommended in such patients [14]. The patient in this case was given spironolactone therapy, which controlled the blood pressure to normal.

In summary, the present study reports the case of a Chinese patient with non-classical 11OHD and presenting with early-onset hypertension, and carrying compound heterozygous mutations in CYP11B1, of which one mutation is reported for the first time. We also analysed and summarised over 170 cases that were previously reported in the literature and found that exons 3 and 8 were mutation hotspots. Mutations in exon 3 often result in non-classical 11OHD, whereas mutations in exon 8 more often result in complete loss of enzymatic activity and classical 11OHD. The possibility of non-classical 11OHD should be considered in hypertensive patients with hyperandrogenism or elevated mineralocorticoids. Such patients should be carefully identified and given an early diagnosis and treatment to avoid the adverse outcomes caused by hyperandrogenism or long-term hypertension.
